# “How can we involve Patients?” - Students’ perspectives on embedding PPIE into a doctoral training centre for AI in medical diagnosis and care

**DOI:** 10.1186/s40900-025-00750-y

**Published:** 2025-07-07

**Authors:** Aron Syversen, Oliver Umney, Lewis Howell, Jack Breen, Emma Briggs, Zoe Hancox, Sobia Khan, Oliver Mills, Victoria Moglia, Mary Paterson, Richard Stephens

**Affiliations:** 1 School of Computer Science, University of Leeds, Leeds, UK; 2 Cancer Research Advocates Forum UK, London, UK

**Keywords:** Patient and public involvement and engagement, Doctoral training programmes, Artificial intelligence, Healthcare, PhD students, Junior researchers

## Abstract

Artificial intelligence (AI) promises to transform healthcare research. However, patients and the public are still not widely involved or engaged within this research area. There is a growing recognition of the importance of incorporating Patient and Public Involvement and Engagement (PPIE) earlier into researcher training. Doctoral training programmes train and support cohorts of PhD students all within a similar research field and therefore may provide the perfect environment to train researchers in PPIE. This paper describes and evaluates the PPIE activities and training within the Centre for Doctoral Training (CDT) in Artificial Intelligence for Medical Diagnosis and Care (“AI-Medical”), at the University of Leeds in the United Kingdom. Authored primarily by PhD candidates from the AI-Medical CDT, it provides an overview of the PPIE activities conducted by students in the CDT between 2021 and 2024. The paper includes first-hand accounts of student experiences, evidenced by quotes, and reflects on these experiences whilst also sharing key learning outcomes. The paper also reflects on the suitability, difficulties, and benefits of including PPIE activities as part of doctoral training programmes, which both develop research leaders of the future and support the students in completing their PhDs. This is particularly important given the current lack of examples incorporating PPIE into AI research projects. It also offers some actionable recommendations for integrating PPIE into future PhD research, whether in other PhD training programmes or within individual research projects. Although written from the viewpoint of the PhD students, this paper will be of interest to patients and the public too, given the increasing use and exploration of AI in health research and therefore the need for the involvement of patients and the public in that work.

## Abstract (unstructured)

Artificial intelligence (AI) in healthcare is a rapidly developing research field, but there is limited evidence that patients and public are widely engaged or involved with its progression. Alongside this, there is a growing recognition of the importance of incorporating Patient and Public Involvement and Engagement (PPIE) earlier into researcher training. Doctoral training programmes (centres) may provide the perfect environment to address both issues. This paper describes and evaluates Patient and Public Involvement and Engagement (PPIE) activities within the Centre for Doctoral Training (CDT) in Artificial Intelligence for Medical Diagnosis and Care (“AI-Medical”), at the University of Leeds in the United Kingdom. Authored primarily by PhD candidates from the AI-Medical CDT, it gives an overview of the PPIE activities conducted within the CDT, including accounts of first-hand experiences, supported by quotes and reflections from students. It also shares key learning outcomes and makes actionable recommendations for integrating PPIE into future PhD programmes and individual research projects. These insights highlight both the successes and challenges of embedding PPIE in healthcare-focused AI research projects in a doctoral training centre.

## Background

Artificial intelligence (AI) encompasses a broad range of technologies that can perform tasks that normally require human-level abilities or beyond. It promises to transform multiple aspects of healthcare, such as diagnosing patients from medical images, providing support to clinicians or uncovering insights into population health [1–3]. However, its use comes with risks and challenges, such as reinforcing inequalities, harming trust in medical practice and risking the privacy of patient data [1, 2, 4]. Involving and engaging patients early in the development of these tools could help to mitigate and increase awareness of these risks [1, 2].

Patient and Public Involvement and Engagement (PPIE) encapsulates both active participation by patients and the public in research (involvement), and sharing and explaining research with patients and the public (engagement) [5]. It is widely accepted that PPIE benefits health research through improved study design, patient outcomes, research dissemination and patient/researcher knowledge and skills [6–9]. There is also a growing recognition of the importance of embedding PPIE earlier into researcher training and development, to help shape future researchers and therefore research [10–13].

Efforts to include PPIE in doctoral training have highlighted numerous challenges posed by the lack of time, money, infrastructure and support [10–13]. Training programmes such as Centres for Doctoral Training (CDTs), which incorporate cohort-based training with the traditional PhD format, may provide the structure and scale required for efficient adoption [14, 15]. Given the large numbers of doctoral training programmes in the UK and the growth in similar course-based doctoral programmes internationally, understanding how to incorporate PPIE into these programmes is becoming increasingly important [15, 16].

This paper provides a students’ perspective of embedding PPIE into a doctoral training programme for students developing AI tools for health research. We highlight the opportunities and challenges of embedding PPIE into a large (~ 50 students) and structured training programme. Further, we reflect on the unique challenges of involving and engaging patients in AI research, which is less widely discussed. We hope this will help future doctoral training programmes planning to embed PPIE.

## Overview of the AI-Medical CDT and its PPIE activities

The Leeds AI-Medical CDT, or the UK Research and Innovation (UKRI) Centre for Doctoral Training in Artificial Intelligence for Medical Diagnosis and Care, is composed of ~ 50 students studying for a PhD at the University of Leeds. Their research projects involve developing AI tools to address different healthcare problems across the themes of screening and early detection, diagnosis, treatment and care. Unlike many traditional PhD programmes, where students propose their own research projects, students choose from a list of projects that are approved by the CDT board. This board includes: a patient representative (RS), who evaluates alignment with patient priorities; academic directors, who assess technical feasibility and innovation; clinical directors, who evaluate clinical relevance and impact; and student representatives, who ensure the projects match students’ research interests.

Students were recruited between 2019 and 2024 and enrolled on a four-year fully funded integrated Master’s and PhD program. The cohort is diverse with a wide range of ages and a near equal gender balance. Students also have a broad range of academic backgrounds, including technical sciences (Computer Science, Mathematics, Engineering, and Physics) and biological or health sciences (Biomedical sciences, Medicine, and Psychology).

We provide a brief overview of the PPIE activities and events organised through the AI-Medical CDT. This includes workshops that trained the students how to do PPIE, engagement activities and involvement activities (summarised in Table 1). Many of the engagement activities conducted within the CDT have considered the public and patients as a general group, whereas involvement activities have focused on specific patient groups (including carers and family members).

### Training in how to do PPIE

Training in how to do PPIE was implemented for all students in the AI-Medical CDT through the *Future Leaders* programme. This taught students how to engage with patients and incorporate patient perspectives and experiences into their projects. For example, all students attended a workshop on written communication of science for non-expert audiences at the beginning of their PhD, as well as workshops on Responsible Research and Innovation (RRI) and co-production sessions with patients and the public. Students have also developed their presentation skills through a series of workshops run by the Exa Foundation and SkillsHouse Bradford [17, 18], focusing on delivering interactive and stimulating content to school-aged children. Several students also engaged with the university’s ‘Be Curious Associates’ and ‘Public Engagement Associates’ programmes, designed to train researchers to effectively present their work to the public, including creating engaging materials, building an accessible online presence, and working with under-served communities. Further, the patient representative (RS) on the CDT board was able to provide valuable mentoring to many of the students (alongside input to their projects) at CDT events.

### Engagement activities with the public

Public engagement has been valuable for personal development (e.g. confidence) and professional development (e.g. improved communication skills). A particularly active area of the CDT’s public engagement has been outreach involving school-aged children and young people. These sessions have been delivered on campus, at local primary and secondary schools, and at science fairs. They have included interactive demonstrations using AI, such as real-time detection of household objects from images, predicting age from voice recordings, and demonstrating how computers can use decision trees to classify images of cats and dogs. These sessions led to valuable discussions about the potential applications and ethical implications of AI in medicine, including bias and fairness. Many of these activities were organised through existing channels within the Science, Technology, Engineering and Mathematics (STEM) outreach team at the University of Leeds or through Skills House Bradford [18]. The CDT students also twice participated in a national outreach programme (In2STEM) [19], hosting groups of 16–19-year-old students for a week-long placement involving planned talks and hands-on workshops applying machine learning to health datasets.

Several students have engaged with the wider community through presentations and discussions (organised independently or through the STEM outreach team at the University of Leeds), including at Café Scientifique and Pint of Science, both of which are well-established national initiatives to engage the public in state-of-the-art scientific research [20, 21]. Presentations have covered a range of topics, from general overviews of AI in medicine to specific talks about fairness in AI, the use of speech in disease detection, and the use of AI in histopathology.

The students have created a blog to share details of their work and publications in an accessible format, events they have attended (conferences and training sessions), and activities they have been involved with (outreach events) (available at: leeds-ai-cdt.github.io). This provides a publicly accessible insight into the type of work conducted within the AI-Medical CDT, as well as ongoing PPIE activities.

### Involvement and engagement activities with patients

Alongside patient involvement in individual research projects, the AI-Medical CDT organised events to allow all students on the training programme the opportunity to involve and engage with patients. To date, the CDT has hosted two major co-production workshops, where doctoral students, patients, and clinicians came together to propose solutions to real-world healthcare problems. Patients were asked to suggest issues they have encountered in healthcare where AI could potentially provide benefit. Each group discussed the context of the problem before identifying key challenges and proposing potential solutions. These were then presented to the wider audience, with active involvement from all parties.

Another example of centrally organised PPIE events is the AI-Medical “Dragon’s Den” event, which has been run twice. In these sessions, students presented a short elevator-pitch-style talk to an online panel of patients from the Cancer Research Advocates Forum (formerly known as the National Cancer Research Institute Consumer Forum), outlining their project in plain English and identifying the potential benefits for patients. The patient panel gave feedback both on the presentation and the individual project, before participating in a whole-group Question & Answer session. Students received feedback on how to present their research more clearly and guided by the priorities of the patients, students were also advised on how they might find further patient input for their projects.

Where possible, the AI-Medical CDT students also worked with existing PPIE networks to simplify the organisation and recruitment of patients and public contributors. For example, a group of four students presented a session to patients at a PPIE meeting held by the Leeds Institute of Clinical Trials Research (LICTR). In the session, the students introduced the concept of AI, and each highlighted an example of how patient data was being used in their research. The students then hosted a structured discussion with patients focusing on the risks and challenges of medical AI research, including how to involve patients effectively.

**Table 1:** Activity table. Summary of the PPIE activities across the CDT programme

Group	Activity name (Activity Type)	Brief description	Key benefits (as identified in this article)
**Patients**	Co-production workshops (Involvement)	Collaborative sessions involving patients, clinicians, and students discussing solutions to real-world healthcare problems.	· Improved communication · Understanding patient experiences
	Dragons’ Den (Involvement)	‘Dragons’ Den’ style elevator pitches to patient representatives, discussion, and feedback.	· Alleviate patient concerns · Feedback on project goals · Improved communication · Information prioritisation
	Discussions with patient groups (e.g. Leeds Institute of Clinical Trials Research session) (Engagement & Involvement)	Presentations on individual research and general topics around AI and the use of patient data. Structured discussions with public and patient contributors.	· Understanding patient involvement · Renewed motivation · Understanding patient concerns · Incorporating patient perspectives · Gain contributors · Realising limitations
	Mentoring from a Patient Representative (Engagement & Involvement)	Mentoring and one-on-one discussions with an experienced patient representative (RS).	· Prioritising patients’ needs · Informing project design · Collaboration · Understanding patient preferences
**Public**	Science fairs and talks (Engagement)	Presentations on AI in Healthcare to the public in relaxed and informal settings, e.g. BeCurious Live, Pint of Science, Café Scientifique.	· Develop presentation and communication skills · Increase public awareness and understanding · Understand public perspective/concerns
	Working with primary and secondary school students (Engagement)	• Talks and workshops for primary and secondary school students, both on campus and in schools, with broad discussions of AI in healthcare. • Hosting A-level students as part of the In2Stem programme.	· Improved communication · Understand concerns of young people · Increase awareness, understanding and skills of young people
	Online public presence (Engagement)	• Student profiles on the University and CDT websites with plain English summaries of research areas. • Student-run blog showcasing research highlights, publications, and team-building and outreach activities.	· Raise awareness · Showcase aims and outputs · Foster transparency and trust · Increase accessibility of research
**Students (training)**	Science communication workshops (Training)	Sessions run by experts in science communication for the public delivering advice, good practice guidelines, and techniques for engaging plain English communication, e.g. Exa Foundation and Skills House Bradford workshops.	· Communication skills · Increased understanding
	RRI workshops (Training)	Set of workshops introducing researchers to the AREA framework and guidelines for RRI [22].	· Reflect on potential implications of research · Application of frameworks to research projects
	Plain English Writing (Training)	Session to focus on writing plain English summaries to support research dissemination.	· Clear writing skills · Avoiding jargon and technical terms

## Reflections

In this section, the students of the AI-Medical CDT reflect on their experiences involving and engaging with patients. Quotes were collected informally from authors, based on their experiences, reflections and discussions about their involvement in PPIE throughout their studies. The student authors are between 24 and 30 years old, are a gender-balanced group and have a mix of professional and educational backgrounds. We include direct quotes from the authors to highlight the impact of PPIE interactions on research, personal development outcomes, and the relationship between researchers and patients. These reflections were collaboratively grouped into three key themes, selected based on recurring ideas and shared experiences.

### Theme 1: impact on students’ research projects

*“Having a patient representative on the board of directors helps to ensure that patient priorities are considered in the projects that are made available to students.” (LH)*.

*“Early engagement from my supervisor with a PPIE group led to shock amongst attendees*,* particularly when they were made aware of gaps in clinical prediction tools*,* such as adding something as simple as time between healthcare visits to models which are hugely valuable predictors. It’s important to consider PPIE early on to ensure knowledge transfer happens both ways.” (ZH*,* feedback from a PPIE event organised by their supervisor).*

*“Involving patients and gaining feedback before beginning data collection would have prevented some of the issues we came across” (AS).*

Students highlighted the importance of timely PPIE to better align their projects with patient needs and to improve their research. Before the students started their projects, the patient representative (RS) helped ensure that shortlisted projects addressed PPIE requirements and focused on improving patient outcomes. Some projects benefited from their supervisors’ experience in conducting PPIE, working with established PPIE groups early in the project development stage. This provided useful disease- or pathway-specific input, helping to ensure the projects were understandable and aligned with real healthcare needs. Failing to incorporate PPIE early in the project sometimes led to avoidable problems and missed opportunities.

*“The priorities of patients and clinicians don’t always match. Often*,* clinicians want to prioritise efficiency*,* whilst patients want to prioritise caution. By involving patients*,* we can ensure that projects cater to both sides.” (MP)*.

*“PPIE has helped me focus my research to not only improve model performance*,* but to make these models explainable to people with different expertise*,* so clinicians and patients can decide whether they trust these models.” (ZH)*.

The students also acknowledged that the priorities of clinicians do not always align with the needs and desires of patients. PPIE was able to help bridge these gaps, ensuring that patient needs are also being met in projects with clinical supervisors.

*“My project involves developing methods for the microscopic analysis of tissue samples. The pathologists who would use these methods are typically not patient-facing*,* making it challenging to understand how PPIE fits within the project.” (JB).*

*“It’s very important to be able to point to PPIE when completing applications for data collection*,* as this is specifically required for HRA applications. However*,* if not done properly*,* it can feel like a box-ticking exercise.” (MP)*.

However, some students expressed concerns that PPIE could become tokenistic. In projects that prioritised technical and theoretical advances, students felt they did not benefit from project-specific PPIE. These included projects developing tools (e.g. novel machine learning algorithms) to assist other researchers in health and biomedicine. These students were concerned that necessitating PPIE would mean patients were involved only superficially without contributing to the research, wasting patients’ time and discouraging them from engaging in future research. This fear of tokenism was also highlighted by students completing ethics applications or approvals that explicitly required PPIE, such as those required for Health Research Authority (HRA) approval.

### Theme 2: researcher and personal development

*“Presenting my projects to patients not only meant I received feedback on the goals of my project*,* but it really highlighted what aspects of the research patients want to know more about. The experience will help me in future to communicate my project more effectively to non-technical audiences*,* by prioritising the most relevant and interesting information for my audience while also managing expectations of the outcomes of my research.” (VM*,* Dragons Den).*

*“I presented my research to the public at an event called Pint of Science. This gave me valuable real-world experience in communicating in plain English and allowed me to apply the skills I had learned at training events.” (JB).*

*“For most of us [as PhD students]*,* talking with patients in a research setting isn’t something we have done before*,* or something we get to do as part of our project. It has made me more confident in this setting and definitely makes me think about how I could involve PPIE in other projects moving forward.” (AS).*

The students identified how PPIE events developed their communication skills and confidence, particularly around communicating their research with non-technical audiences. This included students who felt that PPIE was not a critical part of their research project (JB), but that PPIE training and activities were important opportunities to learn skills that will help them to implement PPIE in future. They also highlighted the importance of training, such as the plain English writing sessions implemented as part of the Future Leaders programme, to fully realise the benefit of these experiences.

*“Before collecting data*,* I contacted some patients to discuss the materials being used. This was facilitated through my clinical supervisor and was very informal (phone conversations). I did not feel I was equipped appropriately to get the most out of this for myself or the patients” (MP).*

*“Although it was daunting discussing data privacy and security with patients due to my limited knowledge and experience*,* actually talking with patients helped me to appreciate their concerns and to alleviate some of them.” (OU*,* Dragons Den session*,* in response to patients’ concerns around data privacy and security risks).*

Several students acknowledged that a lack of appropriate training early in the program meant they felt ill-equipped to maximise the value of the opportunities they had to involve patients. These concerns often related to uncertainty about how to facilitate dialogue, set clear expectations, and manage sensitive discussions. However, they also acknowledged that some skills, such as navigating discussions around complex concerns and issues identified by patients, can only be fully developed through practical experience. Through these discussions, students developed a better understanding of patient concerns and gained confidence in dealing with them.

*“Discussing research with the patients who could benefit directly from it puts the challenges in perspective and is incredibly motivating.”* (LH, LICTR event).

“*Hearing directly from patients about their biggest concerns was refreshing compared to largely technical discussions with supervisors” (AS*, LICTR event*).*

*“Even though my project didn’t require extensive patient involvement*,* learning how to engage with members of the public has been beneficial. In particular*,* speaking to school pupils about AI has helped me to reflect on the impact AI might have on the public*,* such as being challenged about AI taking their future jobs or their parents’ current ones. It has also helped me to appreciate the scale of the challenge to educate and inform the public about what AI is and what its risks are. It has also been a lot of fun!” (OU).*

Engaging with patients and the public helped students to stay motivated and keep sight of the real-world impact of their research. This was particularly valuable to students who felt that the technical challenges of the project sometimes overshadowed the human-motivated benefits. It also challenged their preconceptions of how research impacts patients, the priorities of non-technical stakeholders, and the ethical considerations when applying AI in healthcare. Students also noted how enjoyable engaging with patients and the public could be, often pushing for additional opportunities and events.

### Theme 3: Building and maintaining a dialogue between patients and researchers

*“[There are] PPI/Public contributors [who] would be keen/willing to help in our work if we ask for it”* but “*We are still failing as the AI community to incorporate their (patients’) voices adequately” (LH and OU*, LICTR event*)*.

*“All the interactions I have had with patients and the public discussing my project*,* I have wanted to continue into a longer dialogue*,* [and] having somewhere to signpost them to*,* a website with easy-to-understand project descriptions and ways to reach out to us would have helped this massively” (OU*,* LICTR event).*

The students also noted the difficulties in building and maintaining a long-term dialogue between themselves and the patients beyond one-off PPIE events or individual projects. This in part led to the students creating a blog (https://leeds-ai-cdt.github.io/), which contains plain English summaries of projects and publications from the CDT, as well as reflections on outreach and public engagement activities. Engaging with patients also helped the students to appreciate how much still needs to be done to incorporate the patient voice into medical research using AI, sparking a deep interest in PPIE (and inspiring the authors to write this paper).

*“Describing my project to a patient led them to ask whether the AI model could be applied to other areas*,* such as withdrawal from chemotherapy treatment*,* which is something I hadn’t seen before.” (ZH).*

The students also highlighted the possibility of PPIE leading to new research and benefits beyond the current research projects. This emphasises the impact that PPIE can have on research groups where patients can directly contribute to the design of research.

## Discussion

In this paper, PhD students reflected on their experience of incorporating PPIE into a doctoral training programme for research into AI within healthcare. The students highlighted the impact on their research projects (Theme 1), their own professional and personal development (Theme 2) and how they contributed to a longer-term dialogue between patients and researchers (Theme 3). They reflected on the many benefits of having centralised support through the CDT, helping to ensure PPIE was embedded from project conception to completion, improved students’ confidence and communication skills, and provided the opportunity to learn through co-production events with patients as partners in research. They also acknowledged the challenges and identified areas for improvement that future doctoral training programmes may be well placed to address.

Several students highlighted how patient insight and experiences led to high-quality research in their projects. Timely implementation of PPIE informed data collection, project focus and flagged issues early into project development (Theme 1). However, this often depended on the level of experience and interest held by the project supervisor, meaning not all students benefited equally. Further, several challenges were reported, including missed opportunities for PPIE in the early stages, while other students reported fears of tokenistic involvement in highly technical projects, further from application into healthcare practice. This shows that despite the benefits provided by a centrally organised CDT, individual projects still require a tailored approach. This could have been mitigated by involving a broader range of patients on the CDT board that vetted the project proposals. Involvement at this stage can help to guide the PPIE timing and requirements for each project, ensuring the appropriate support is in place before the project begins. However, not all benefits of PPIE were seen through direct impact on research.

Incorporating PPIE at an early stage in a researcher’s training can help their personal and professional development and ensure patient perspectives are embedded into their future approach to research. Participating in co-production workshops and patient engagement activities improved the students’ communication skills, particularly when conveying complex technical ideas to non-specialist audiences. This is particularly relevant to AI research, where public understanding and trust are key to the development and adoption of new technologies. These experiences also contributed to a stronger sense of purpose, connecting students to the real-world impact of their work, which could help to address feelings of isolation and disillusionment that may contribute to high dropout rates in doctoral programs [23].

Although CDTs have a longer life span than individual PhD projects, they still have a fixed term, and so ensuring the impact of PPIE extends beyond the program’s conclusion remains a challenge. Collaborating with established internal (e.g. university departments) and external groups (e.g. patient groups) or opening up CDT events beyond the CDT cohort could help start longer-term projects. This reflects the experiences of the students, who identified that collaborative events often sparked a genuine desire for ongoing involvement among both students and patients. For example, establishing public-facing conferences that require the use of plain English in posters, abstracts, and presentations could allow a wider group of students to practice communication of their scientific work with non-technical audiences.

The success of AI within healthcare depends on fostering trust and collaboration among all stakeholders. As the end-users of healthcare technologies, patient perspectives are crucial to ensure that AI solutions are patient-centred and don’t reinforce existing inequalities [24]. In comparison with the standard PhD model, CDTs offer an advantageous structure to support PPIE in AI research due to shared funding and central management boards for planning and hosting events. These programmes allow PPIE to be embedded into the training of a researcher from the earliest stage, developing the next generation of responsible and socially aware scientists. This will, in turn, help to develop solutions to healthcare problems that align with societal needs, helping to realise the public’s investment in healthcare and AI research.

Based on the above reflections, we present recommendations which we hope will serve as a useful starting point for future doctoral training programmes and research groups wanting to involve patients in health data research (Table 2).

**Table 2:** Recommendations for embedding PPIE into doctoral training programmes

	Recommendations	Themes addressed
**Organisation of doctoral training programmes**	Require consideration of PPIE for all project proposals.	1
	Encourage projects designed in co-production with patients or that have involved patients in the conceptualisation.	1
	Have a diverse PPIE panel to review all project proposals to assess PPIE requirements and potential impact.	1
	Consider a ‘patient star of approval’ system, or similar, to allow students to make informed decisions regarding patient impact when selecting PhD projects.	1
	Provide specialist support to address project-specific PPIE requirements when regular patient input is required. This could include training, assistance in identifying appropriate patient groups or involving patients in a supervisory role.	1
	Offer PPIE training early in the PhD training programme to ensure students have sufficient time to engage in PPIE and implement changes guided by participants.	1, 2
	Require students to attend compulsory training and engagement events, highlighting the opportunities to bolster research skills. Training events should be organised early into the doctoral programme, including effective PPIE, plain English (non-technical) writing, and RRI.	2
	Require students to write plain English summaries for their project and for publications resulting from their work.	2
	Invite patients and public contributors to attend internal academic events such as conferences, to discuss projects and see the impact their input has had. Posters and talks must be made accessible to the audience.	1, 3
	Assign an experienced member of staff and a budget to PPIE support and event planning to reduce the reliance on individual supervisor experience for organising PPIE activities.	1
	Maintain a website documenting public-facing activities, student profiles, project summaries and plain English summaries of all publications that patients and the public can easily access.	3
	Create communication channels for student representatives to engage with the doctoral programme management board, providing feedback on challenges around PPIE.	1,2
**Conducting PPIE events**	Ensure that contributions of patients and the public involved in research are acknowledged in line with PPIE practice. For example, students should appropriately credit the use of patients’ data in their research through the ‘useMYdata’ patient citation, easily encouraged by having readily available stickers [25].	1
	Be transparent with patients about the specific areas where their input will have an impact, ensuring their involvement is meaningful and avoids tokenism.	1, 3
	Engage with established PPIE groups and networks to ensure that relevant patient groups are involved.	1, 3
	Where possible, coordinate with existing local events, groups, and activities, both to involve the local community and ensure enduring connections are formed during the training programme.	3

## Conclusion

This paper discussed and reflected on the experiences of PhD students learning and practising PPIE within the Leeds AI-Medical CDT. These experiences have been predominantly positive for both the students’ professional and personal development and for enhancing ongoing research. Reflecting on the challenges posed by embedding PPIE within the CDT has also allowed us to consider what we would do differently if we had the opportunity to start afresh. This forms the basis of our recommendations for future doctoral training programmes.

## Data Availability

No datasets were generated or analysed during the current study.

## Abbreviations

UKRI: United Kingdom Research and Innovation
CDT: Centre for Doctoral Training
AI-Medical: Artificial Intelligence for Medical Diagnosis and Care
AI: Artificial Intelligence
PPIE: Patient and Public Involvement and Engagement
RRI: Responsible Research and Innovation
STEM: Science Technology Engineering and Maths
HRA: Health Research Authority
LICTR: Leeds Institute of Clinical Trials Research

## References

[1] Rajpurkar P, et al. AI in health and medicine. Nat Med. 2022;28(1):31–8. 35058619 10.1038/s41591-021-01614-0

[2] Alowais SA, et al. Revolutionizing healthcare: the role of artificial intelligence in clinical practice. BMC Med Educ. 2023;23(1):689. 37740191 10.1186/s12909-023-04698-z PMC10517477

[3] Beam AL, et al. Artificial intelligence in medicine. N Engl J Med. 2023;388(13):1220–1. 36988598 10.1056/NEJMe2206291

[4] Carey S, Pang A, Kamps M. Fairness in AI for healthcare. Future Healthc J. 2024;11(3):100177. 39371535 10.1016/j.fhj.2024.100177 PMC11452831

[5] Tarpey HHSBM. *Briefing notes for researchers - public involvement in NHS, health and social care research*. 2012 [cited 2024 14th December]; Available from: https://www.nihr.ac.uk/briefing-notes-researchers-public-involvement-nhs-health-and-social-care-research

[6] Brett J, et al. Mapping the impact of patient and public involvement on health and social care research: a systematic review. Health Expect. 2014;17(5):637–50. 22809132 10.1111/j.1369-7625.2012.00795.x PMC5060910

[7] Holmes L, et al. Innovating public engagement and patient involvement through strategic collaboration and practice. Res Involv Engagem. 2019;5:30. 31646001 10.1186/s40900-019-0160-4 PMC6802177

[8] Agyei-Manu E, et al. The benefits, challenges, and best practice for patient and public involvement in evidence synthesis: A systematic review and thematic synthesis. Health Expect. 2023;26(4):1436–52. 37260191 10.1111/hex.13787 PMC10349234

[9] Aiyegbusi OL, et al. Considerations for patient and public involvement and engagement in health research. Nat Med. 2023;29(8):1922–9. 37474660 10.1038/s41591-023-02445-x

[10] Tomlinson J, et al. Patient and public involvement in designing and conducting doctoral research: the whys and the hows. Res Involv Engagem. 2019;5:23. 31428458 10.1186/s40900-019-0155-1 PMC6697942

[11] Coupe N, Mathieson A. Patient and public involvement in doctoral research: impact, resources and recommendations. Health Expect. 2020;23(1):125–36. 31613049 10.1111/hex.12976 PMC6978853

[12] Dawson S, et al. Patient and public involvement in doctoral research: reflections and experiences of the PPI contributors and researcher. Res Involv Engagem. 2020;6:23. 32426162 10.1186/s40900-020-00201-w PMC7216324

[13] Karlsson AW. A. Janssens 2023 Patient and public involvement and engagement (PPIE) in healthcare education and thesis work: the first step towards PPIE knowledgeable healthcare professionals. BMJ Open 13 1 e067588. 36604125 10.1136/bmjopen-2022-067588 PMC9827239

[14] UK Research and Innovation. Review of EPSRC-funded Doctoral Education. 2021 [cited 2025 21st March]; Available from: https://www.ukri.org/wp-content/uploads/2021/10/EPSRC-071021-ReviewDoctoralEducationSupport.pdf

[15] UK Research and Innovation. Centres for Doctoral Training– EPSRC 2023 [cited 2025. 2nd June]; Available from: https://www.ukri.org/what-we-do/developing-people-and-skills/epsrc/studentships/centres-for-doctoral-training/

[16] Yudkevich M, Altbach PG, Wit Hd. Trends and issues in doctoral education: A global perspective. New Delhi: SAGE Publications Pvt Ltd: Mathura Road; 2020.

[17] The Exa Foundation. 2024 [cited 2024 16th December]; Available from: https://exa.net.uk/foundation/

[18] SkillsHouse B. 2024 [cited 2024 16th December]; Available from: https://www.skillshouse.co.uk/

[19] In2STEM. 2024 [cited 2024 16th December]; Available from: https://in2scienceuk.org/our-programmes/in2stem/

[20] Grand A. Cafe Scientifique Sci Prog. 2014;97(Pt 3):275–8. 10.3184/003685014X14098305289149 PMC10365456 25549411

[21] Pint of Science. 2024 [cited 2024 14th December]; Available from: https://pintofscience.co.uk/

[22] UK Research and Innovation. Framework for Responsible Research and Innovation. 2024 [cited 2025 27th March]; Available from: https://www.ukri.org/who-we-are/epsrc/our-policies-and-standards/framework-for-responsible-innovation/

[23] DiscoverPhDs. PhD failure rate– A look at the statistics. 2024 [cited 2024 14th December]; Available from: https://www.discoverphds.com/advice/doing/phd-failure-rate

[24] Lammons W, et al. Centering public perceptions on translating AI into clinical practice: patient and public involvement and engagement consultation focus group study. J Med Internet Res. 2023;25:e49303. 37751234 10.2196/49303 PMC10565616

[25] useMYData. *The Patient Data Citation* 2025 [cited 2025 26th March]; Available from: https://www.usemydata.org/citation.php

